# Back to the Future with Osteotomies around the Hip

**DOI:** 10.3390/jcm11154446

**Published:** 2022-07-30

**Authors:** Sufian S. Ahmad, Henning Windhagen, Vikas Khanduja

**Affiliations:** 1 Orthopaedic Department, Medical School of Hannover, Annastift Hospital, 30625 Hannover, Germany; henning.windhagen@diakovere.de; 2 Addenbrooke’s–Cambridge University Hospital, Cambridge CB2 0QQ, UK; vk279@cam.ac.uk

If we were to look back at the history of orthopedics only two generations ago, the intertrochanteric osteotomy was a well-established procedure for the treatment of osteoarthritis of the hip [1]. The procedure was well approved as a surgical modality with good short- and long-term outcomes, especially in relieving pain [1,2]. However, the introduction of arthroplasty depreciated the value of the osteotomy procedure, and rightly so in the majority of arthritic cases. This resulted in a rapid loss of interest and a decline in the demand for continuing education in that area of expertise. A significant amount of support and thrust from the arthroplasty industry and the commercial interest also led to the decline in the concept of osteotomies. The excellent outcomes and functional gains with a return to an almost normal lifestyle following a total hip arthroplasty (THA) led to the definition of a new, effective and reproducible intervention for the treatment of osteoarthritis of the hip [3].

Proximal femur osteotomies were mainly reserved for selected pediatric orthopedic indications, leaving the majority of modern adult reconstructive surgeons unfamiliar with the technique and its pitfalls. THA thus became the “bread & butter” procedure for the adult reconstruction surgeon.

With total hip arthroplasty on the rise and the corresponding huge focus on refining implants and surgical techniques, there was still enough space for the birth of the niche of hip preservation surgery. This field emerged after a publication by Reinhold Ganz and his colleagues in 2003 [4], describing the idea that cartilage damage is likely to be secondary to a pathomorphology that depicts the mechanistic concept of femoroacetabular impingement (FAI). The exponential growth in publications on hip preservation surgery allowed for further improved understanding of the mechanistic influence of pathomorphology on hip pain and the development of osteoarthritis.

With the current improved understanding of morphology and pathology, a revival of the periacetabular osteotomy (PAO) and proximal femur osteotomies is on the rise and in demand, more than any time before [5].

The fact that reduced femoral antetorsion causes an early anterior conflict upon internal rotation of the hip has raised the significance of appreciating torsional correction in cases where cam and pincer correction alone would result in an inadequate restoration of internal rotation [6]. On the other hand, high femoral antetorsion has been described as being associated with posterior impingement and, in particular, extra-articular impingement between the femur and the ischium [7]. It is also well known that over 1/3rd of the patients with symptomatic FAI have a version or rotational abnormality which requires attention prior to planning any procedure involving the hip itself [6].

The robust method of tackling such pathologies is by working on correcting the underlying morphological abnormality. This mandates sweeping the dust off the stored instrument sets of angled blade plates that are likely to still be found in the cellars of most orthopedic departments. The coming generation of young adult hip surgeons will have to revive the techniques of osteotomy and possibly initiate implant innovation and precision surgery in the field.

On the acetabular side, anterosuperior undercoverage is no longer the only indication for reorientation. The understanding of three-dimensional anatomy underlines the problem of posterolateral undercoverage in a truly retroverted acetabulum as well as focal anterior undercoverage in an anteverted acetabulum with sufficient posterolateral coverage. This allows a wide range of possible variants of pathomorphology that require individualized treatment. With the improvement in safety and efficacy of reorientation procedures, such as the PAO, the threshold for performing surgery is also lowering, allowing many more patients to benefit from the power of its success. Digital technology will aid in this development by driving improvement in the simulation of pathology, surgical planning, and accuracy of concept implementation.

The ideal future hip surgeon will encompass a diverse set of surgical skills that are utilized on an individual patient-specific basis after meticulous analysis of individual pathomorphology based on evidence and digital assistance. That said, the future of hip surgery is certainly exciting and bright!
